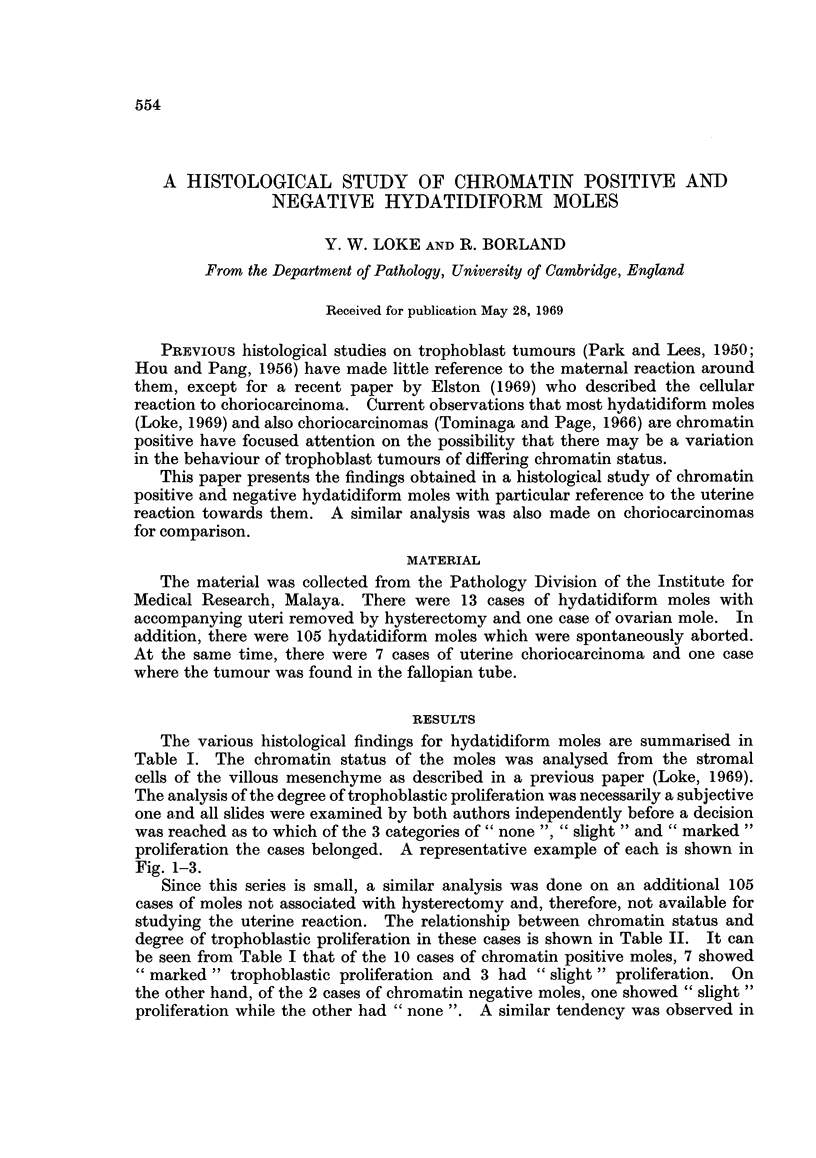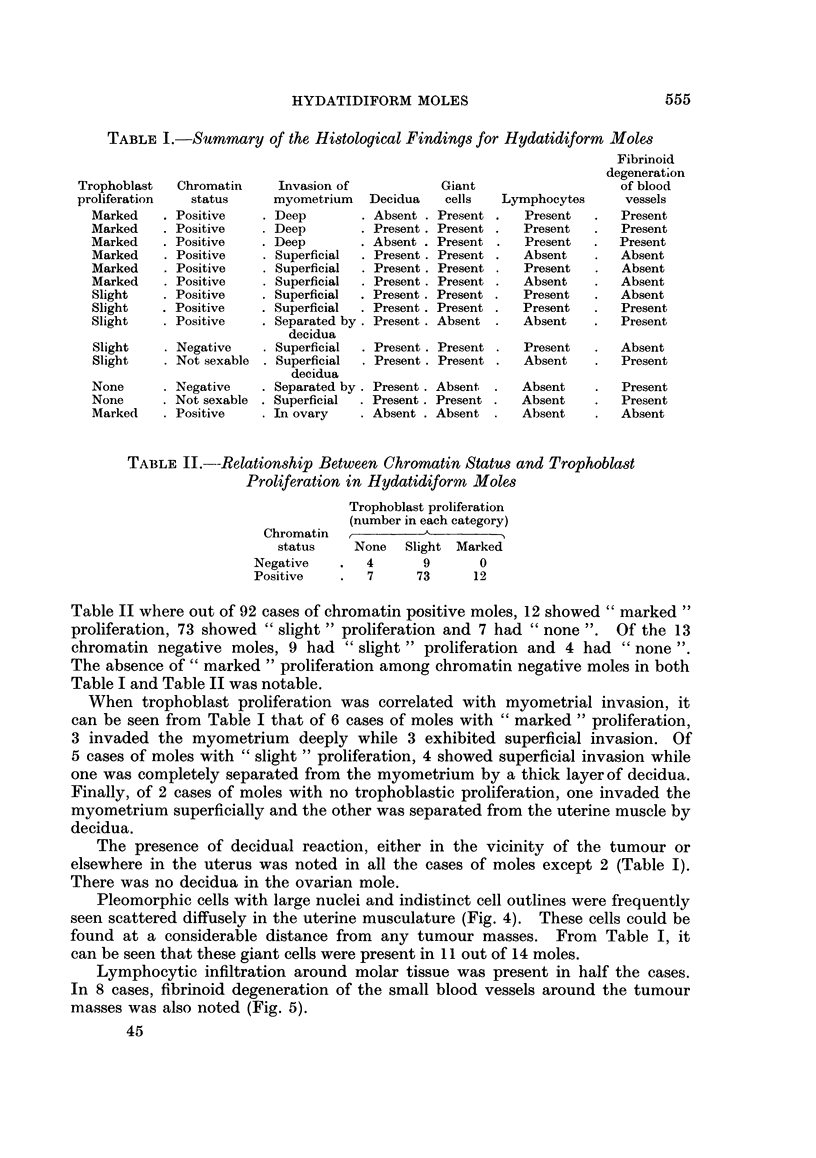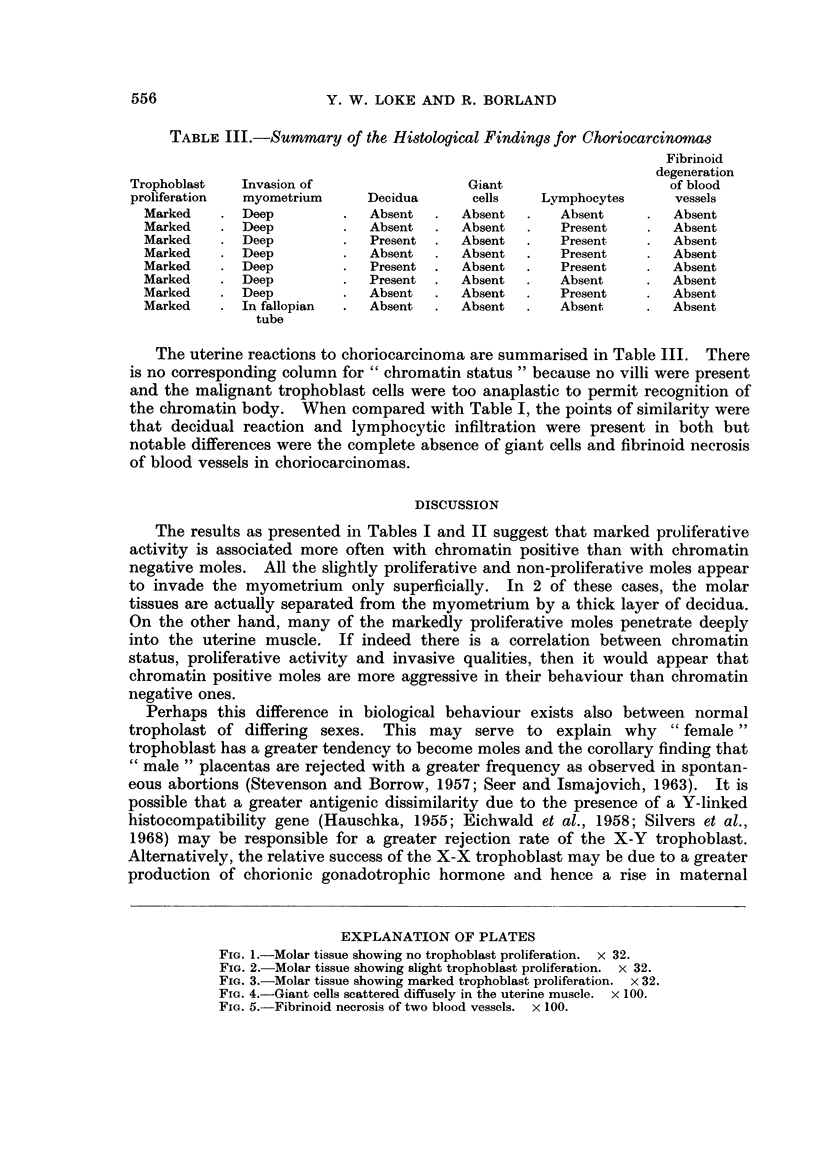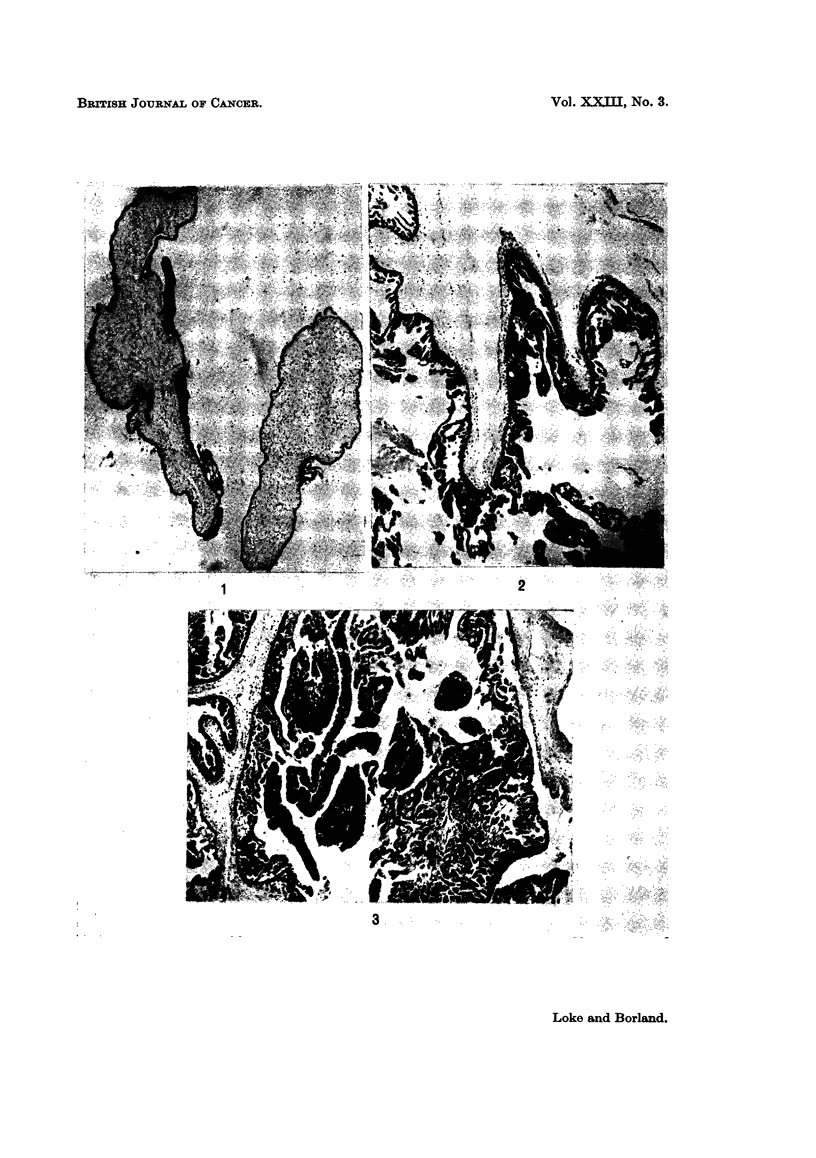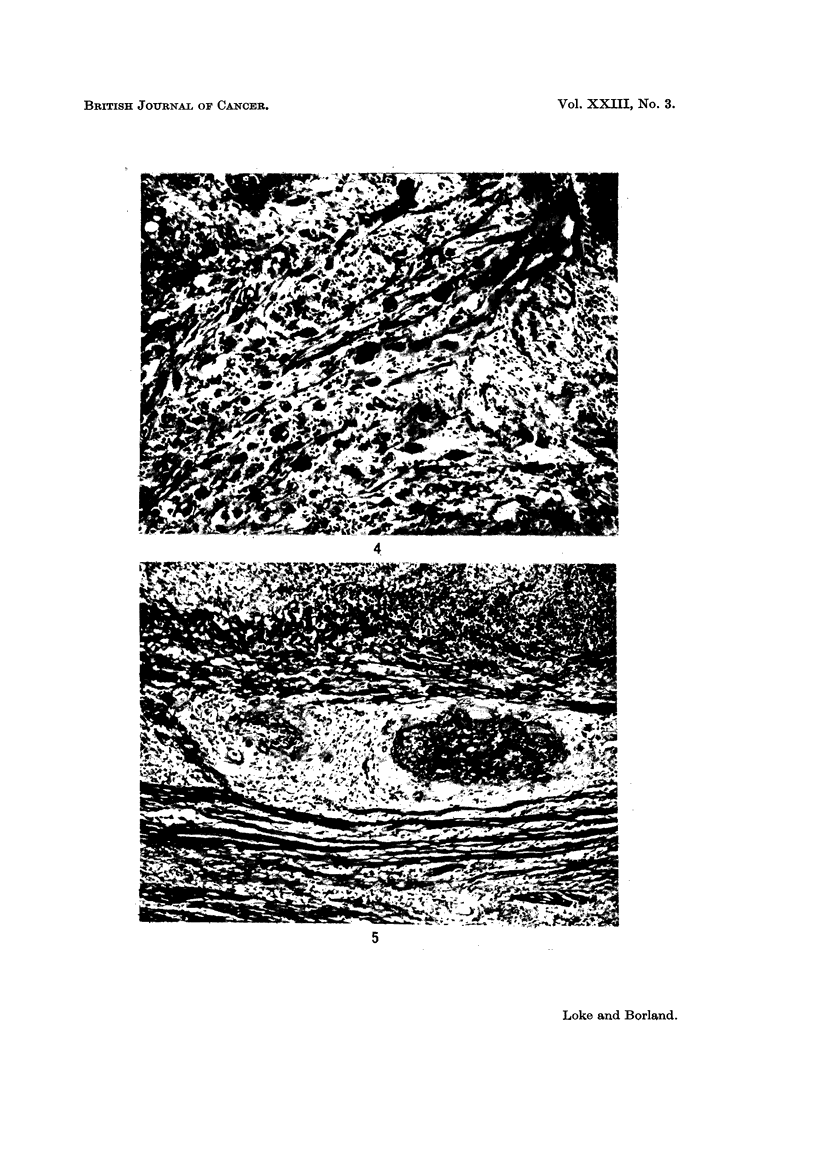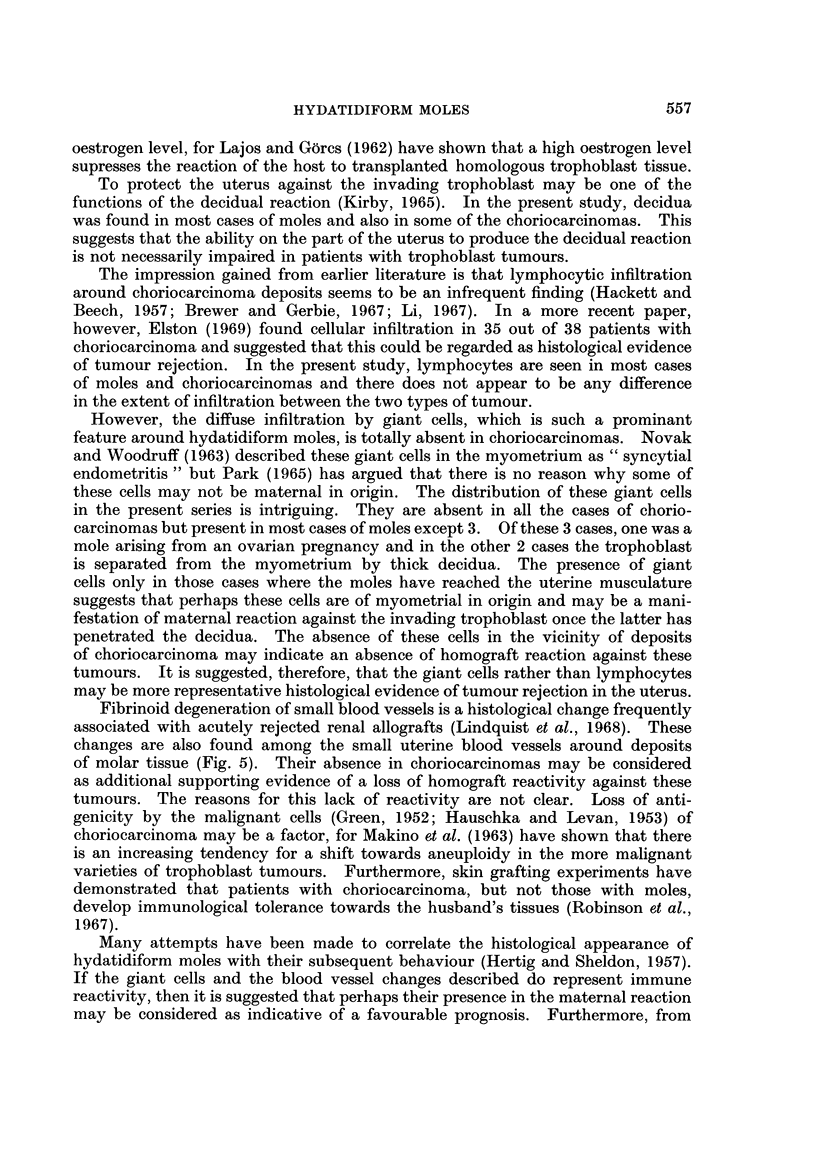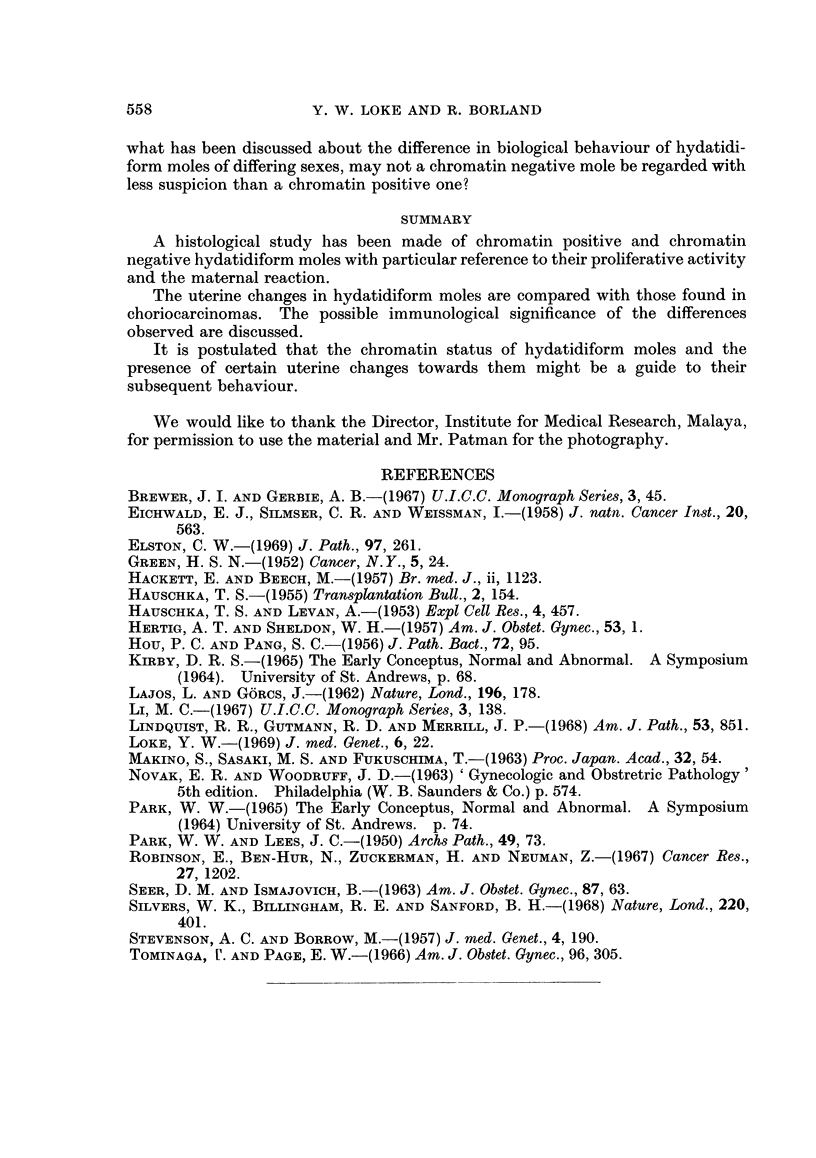# A histological study of chromatin positive and negative hydatidiform moles.

**DOI:** 10.1038/bjc.1969.68

**Published:** 1969-09

**Authors:** Y. W. Loke, R. Borland

## Abstract

**Images:**


### A HISTOLOGICAL STUDY OF CHROMATIN POSITIVE AND NEGATIVE HYDATIDIFORM MOLES

Y. W. LOKE AND R. BORLAND

*From the Department of Pathology, University of Cambridge, England*

Received for publication May 28, 1969

PREVIOUS histological studies on trophoblast tumours (Park and Lees, 1950; Hou and Pang, 1956) have made little reference to the maternal reaction around them, except for a recent paper by Elston (1969) who described the cellular reaction to choriocarcinoma. Current observations that most hydatidiform moles (Loke, 1969) and also choriocarcinomas (Tominaga and Page, 1966) are chromatin positive have focused attention on the possibility that there may be a variation in the behaviour of trophoblast tumours of differing chromatin status.

This paper presents the findings obtained in a histological study of chromatin positive and negative hydatidiform moles with particular reference to the uterine reaction towards them. A similar analysis was also made on choriocarcinomas for comparison.

### MATERIAL

The material was collected from the Pathology Division of the Institute for Medical Research, Malaya. There were 13 cases of hydatidiform moles with accompanying uteri removed by hysterectomy and one case of ovarian mole. In addition, there were 105 hydatidiform moles which were spontaneously aborted. At the same time, there were 7 cases of uterine choriocarcinoma and one case where the tumour was found in the fallopian tube.

### RESULTS

The various histological findings for hydatidiform moles are summarised in Table I. The chromatin status of the moles was analysed from the stromal cells of the villous mesenchyme as described in a previous paper (Loke, 1969). The analysis of the degree of trophoblastic proliferation was necessarily a subjective one and all slides were examined by both authors independently before a decision was reached as to which of the 3 categories of " none ", " slight " and " marked " proliferation the cases belonged. A representative example of each is shown in Fig. 1–3.

Since this series is small, a similar analysis was done on an additional 105 cases of moles not associated with hysterectomy and, therefore, not available for studying the uterine reaction. The relationship between chromatin status and degree of trophoblastic proliferation in these cases is shown in Table II. It can be seen from Table I that of the 10 cases of chromatin positive moles, 7 showed " marked " trophoblastic proliferation and 3 had " slight " proliferation. On the other hand, of the 2 cases of chromatin negative moles, one showed " slight " proliferation while the other had " none ". A similar tendency was observed in

TABLE I.—*Summary of the Histological Findings for Hydatidiform Moles*

| Trophoblast proliferation | Chromatin status | Invasion of myometrium | Decidua | Giant cells | Lymphocytes | Fibrinoid degeneration of blood vessels |
|---|---|---|---|---|---|---|
| Marked | Positive | Deep | Absent | Present | Present | Present |
| Marked | Positive | Deep | Present | Present | Present | Present |
| Marked | Positive | Deep | Absent | Present | Present | Present |
| Marked | Positive | Superficial | Present | Present | Absent | Absent |
| Marked | Positive | Superficial | Present | Present | Present | Absent |
| Marked | Positive | Superficial | Present | Present | Absent | Absent |
| Slight | Positive | Superficial | Present | Present | Present | Absent |
| Slight | Positive | Superficial | Present | Present | Present | Present |
| Slight | Positive | Separated by decidua | Present | Absent | Absent | Present |
| Slight | Negative | Superficial | Present | Present | Present | Absent |
| Slight | Not sexable | Superficial decidua | Present | Present | Absent | Present |
| None | Negative | Separated by | Present | Absent | Absent | Present |
| None | Not sexable | Superficial | Present | Present | Absent | Present |
| Marked | Positive | In ovary | Absent | Absent | Absent | Absent |

TABLE II.—*Relationship Between Chromatin Status and Trophoblast Proliferation in Hydatidiform Moles*

| Chromatin status | Trophoblast proliferation (number in each category) | | |
|---|---|---|---|
| | None | Slight | Marked |
| Negative | 4 | 9 | 0 |
| Positive | 7 | 73 | 12 |

Table II where out of 92 cases of chromatin positive moles, 12 showed " marked " proliferation, 73 showed " slight " proliferation and 7 had " none ". Of the 13 chromatin negative moles, 9 had " slight " proliferation and 4 had " none ". The absence of " marked " proliferation among chromatin negative moles in both Table I and Table II was notable.

When trophoblast proliferation was correlated with myometrial invasion, it can be seen from Table I that of 6 cases of moles with " marked " proliferation, 3 invaded the myometrium deeply while 3 exhibited superficial invasion. Of 5 cases of moles with " slight " proliferation, 4 showed superficial invasion while one was completely separated from the myometrium by a thick layer of decidua. Finally, of 2 cases of moles with no trophoblastic proliferation, one invaded the myometrium superficially and the other was separated from the uterine muscle by decidua.

The presence of decidual reaction, either in the vicinity of the tumour or elsewhere in the uterus was noted in all the cases of moles except 2 (Table I). There was no decidua in the ovarian mole.

Pleomorphic cells with large nuclei and indistinct cell outlines were frequently seen scattered diffusely in the uterine musculature (Fig. 4). These cells could be found at a considerable distance from any tumour masses. From Table I, it can be seen that these giant cells were present in 11 out of 14 moles.

Lymphocytic infiltration around molar tissue was present in half the cases. In 8 cases, fibrinoid degeneration of the small blood vessels around the tumour masses was also noted (Fig. 5).

TABLE III.—*Summary of the Histological Findings for Choriocarcinomas*

| Trophoblast proliferation | Invasion of myometrium | Decidua | Giant cells | Lymphocytes | Fibrinoid degeneration of blood vessels |
|---|---|---|---|---|---|
| Marked | Deep | Absent | Absent | Absent | Absent |
| Marked | Deep | Absent | Absent | Present | Absent |
| Marked | Deep | Present | Absent | Present | Absent |
| Marked | Deep | Absent | Absent | Present | Absent |
| Marked | Deep | Present | Absent | Present | Absent |
| Marked | Deep | Present | Absent | Absent | Absent |
| Marked | Deep | Absent | Absent | Present | Absent |
| Marked | In fallopian tube | Absent | Absent | Absent | Absent |

The uterine reactions to choriocarcinoma are summarised in Table III. There is no corresponding column for " chromatin status " because no villi were present and the malignant trophoblast cells were too anaplastic to permit recognition of the chromatin body. When compared with Table I, the points of similarity were that decidual reaction and lymphocytic infiltration were present in both but notable differences were the complete absence of giant cells and fibrinoid necrosis of blood vessels in choriocarcinomas.

### DISCUSSION

The results as presented in Tables I and II suggest that marked proliferative activity is associated more often with chromatin positive than with chromatin negative moles. All the slightly proliferative and non-proliferative moles appear to invade the myometrium only superficially. In 2 of these cases, the molar tissues are actually separated from the myometrium by a thick layer of decidua. On the other hand, many of the markedly proliferative moles penetrate deeply into the uterine muscle. If indeed there is a correlation between chromatin status, proliferative activity and invasive qualities, then it would appear that chromatin positive moles are more aggressive in their behaviour than chromatin negative ones.

Perhaps this difference in biological behaviour exists also between normal tropholast of differing sexes. This may serve to explain why " female " trophoblast has a greater tendency to become moles and the corollary finding that " male " placentas are rejected with a greater frequency as observed in spontaneous abortions (Stevenson and Borrow, 1957; Seer and Ismajovich, 1963). It is possible that a greater antigenic dissimilarity due to the presence of a Y-linked histocompatibility gene (Hauschka, 1955; Eichwald *et al.*, 1958; Silvers *et al.*, 1968) may be responsible for a greater rejection rate of the X-Y trophoblast. Alternatively, the relative success of the X-X trophoblast may be due to a greater production of chorionic gonadotrophic hormone and hence a rise in maternal

EXPLANATION OF PLATES

FIG. 1.—Molar tissue showing no trophoblast proliferation. × 32.
FIG. 2.—Molar tissue showing slight trophoblast proliferation. × 32.
FIG. 3.—Molar tissue showing marked trophoblast proliferation. × 32.
FIG. 4.—Giant cells scattered diffusely in the uterine muscle. × 100.
FIG. 5.—Fibrinoid necrosis of two blood vessels. × 100.

1 2 3

4

5

Loke and Borland.

oestrogen level, for Lajos and Görcs (1962) have shown that a high oestrogen level supresses the reaction of the host to transplanted homologous trophoblast tissue.

To protect the uterus against the invading trophoblast may be one of the functions of the decidual reaction (Kirby, 1965). In the present study, decidua was found in most cases of moles and also in some of the choriocarcinomas. This suggests that the ability on the part of the uterus to produce the decidual reaction is not necessarily impaired in patients with trophoblast tumours.

The impression gained from earlier literature is that lymphocytic infiltration around choriocarcinoma deposits seems to be an infrequent finding (Hackett and Beech, 1957; Brewer and Gerbie, 1967; Li, 1967). In a more recent paper, however, Elston (1969) found cellular infiltration in 35 out of 38 patients with choriocarcinoma and suggested that this could be regarded as histological evidence of tumour rejection. In the present study, lymphocytes are seen in most cases of moles and choriocarcinomas and there does not appear to be any difference in the extent of infiltration between the two types of tumour.

However, the diffuse infiltration by giant cells, which is such a prominant feature around hydatidiform moles, is totally absent in choriocarcinomas. Novak and Woodruff (1963) described these giant cells in the myometrium as " syncytial endometritis " but Park (1965) has argued that there is no reason why some of these cells may not be maternal in origin. The distribution of these giant cells in the present series is intriguing. They are absent in all the cases of choriocarcinomas but present in most cases of moles except 3. Of these 3 cases, one was a mole arising from an ovarian pregnancy and in the other 2 cases the trophoblast is separated from the myometrium by thick decidua. The presence of giant cells only in those cases where the moles have reached the uterine musculature suggests that perhaps these cells are of myometrial in origin and may be a manifestation of maternal reaction against the invading trophoblast once the latter has penetrated the decidua. The absence of these cells in the vicinity of deposits of choriocarcinoma may indicate an absence of homograft reaction against these tumours. It is suggested, therefore, that the giant cells rather than lymphocytes may be more representative histological evidence of tumour rejection in the uterus.

Fibrinoid degeneration of small blood vessels is a histological change frequently associated with acutely rejected renal allografts (Lindquist *et al.*, 1968). These changes are also found among the small uterine blood vessels around deposits of molar tissue (Fig. 5). Their absence in choriocarcinomas may be considered as additional supporting evidence of a loss of homograft reactivity against these tumours. The reasons for this lack of reactivity are not clear. Loss of antigenicity by the malignant cells (Green, 1952; Hauschka and Levan, 1953) of choriocarcinoma may be a factor, for Makino *et al.* (1963) have shown that there is an increasing tendency for a shift towards aneuploidy in the more malignant varieties of trophoblast tumours. Furthermore, skin grafting experiments have demonstrated that patients with choriocarcinoma, but not those with moles, develop immunological tolerance towards the husband's tissues (Robinson *et al.*, 1967).

Many attempts have been made to correlate the histological appearance of hydatidiform moles with their subsequent behaviour (Hertig and Sheldon, 1957). If the giant cells and the blood vessel changes described do represent immune reactivity, then it is suggested that perhaps their presence in the maternal reaction may be considered as indicative of a favourable prognosis. Furthermore, from what has been discussed about the difference in biological behaviour of hydatidiform moles of differing sexes, may not a chromatin negative mole be regarded with less suspicion than a chromatin positive one?

### SUMMARY

A histological study has been made of chromatin positive and chromatin negative hydatidiform moles with particular reference to their proliferative activity and the maternal reaction.

The uterine changes in hydatidiform moles are compared with those found in choriocarcinomas. The possible immunological significance of the differences observed are discussed.

It is postulated that the chromatin status of hydatidiform moles and the presence of certain uterine changes towards them might be a guide to their subsequent behaviour.

We would like to thank the Director, Institute for Medical Research, Malaya, for permission to use the material and Mr. Patman for the photography.

### REFERENCES

Brewer, J. I. and Gerbie, A. B.—(1967) *U.I.C.C. Monograph Series*, **3**, 45.
Eichwald, E. J., Silmser, C. R. and Weissman, I.—(1958) *J. natn. Cancer Inst.*, **20**, 563.
Elston, C. W.—(1969) *J. Path.*, **97**, 261.
Green, H. S. N.—(1952) *Cancer, N.Y.*, **5**, 24.
Hackett, E. and Beech, M.—(1957) *Br. med. J.*, ii, 1123.
Hauschka, T. S.—(1955) *Transplantation Bull.*, **2**, 154.
Hauschka, T. S. and Levan, A.—(1953) *Expl Cell Res.*, **4**, 457.
Hertig, A. T. and Sheldon, W. H.—(1957) *Am. J. Obstet. Gynec.*, **53**, 1.
Hou, P. C. and Pang, S. C.—(1956) *J. Path. Bact.*, **72**, 95.
Kirby, D. R. S.—(1965) The Early Conceptus, Normal and Abnormal. A Symposium (1964). University of St. Andrews, p. 68.
Lajos, L. and Görcs, J.—(1962) *Nature, Lond.*, **196**, 178.
Li, M. C.—(1967) *U.I.C.C. Monograph Series*, **3**, 138.
Lindquist, R. R., Gutmann, R. D. and Merrill, J. P.—(1968) *Am. J. Path.*, **53**, 851.
Loke, Y. W.—(1969) *J. med. Genet.*, **6**, 22.
Makino, S., Sasaki, M. S. and Fukuschima, T.—(1963) *Proc. Japan. Acad.*, **32**, 54.
Novak, E. R. and Woodruff, J. D.—(1963) ' Gynecologic and Obstretric Pathology ' 5th edition. Philadelphia (W. B. Saunders & Co.) p. 574.
Park, W. W.—(1965) The Early Conceptus, Normal and Abnormal. A Symposium (1964) University of St. Andrews. p. 74.
Park, W. W. and Lees, J. C.—(1950) *Archs Path.*, **49**, 73.
Robinson, E., Ben-Hur, N., Zuckerman, H. and Neuman, Z.—(1967) *Cancer Res.*, **27**, 1202.
Seer, D. M. and Ismajovich, B.—(1963) *Am. J. Obstet. Gynec.*, **87**, 63.
Silvers, W. K., Billingham, R. E. and Sanford, B. H.—(1968) *Nature, Lond.*, **220**, 401.
Stevenson, A. C. and Borrow, M.—(1957) *J. med. Genet.*, **4**, 190.
Tominaga, [illegible]. and Page, E. W.—(1966) *Am. J. Obstet. Gynec.*, 96, 305.